# 54 Managing Edema, Pain, and Mobility in Burn Patients with Short Stretch Compression Bandaging: Case Series

**DOI:** 10.1093/jbcr/iraf019.054

**Published:** 2025-04-01

**Authors:** Cody Thornburg, Andria Martinez, Derek Murray, Karen Richey, Kevin Foster

**Affiliations:** Diane & Bruce Halle Arizona Burn Center; Diane & Bruce Halle Arizona Burn Center; Diane & Bruce Halle Arizona Burn Center; Diane & Bruce Halle Arizona Burn Center; Diane & Bruce Halle Arizona Burn Center

## Abstract

**Introduction:**

Application of compression bandages is standard in pain and edema management in the acute burn setting. Long stretch (LS) elastic bandage, is the bandage of choice amongst burn centers. LS bandage is known for its high elasticity which demonstrates a high resting pressure and low working pressure. LS compression, typically used for acute edema management, can also be utilized for improved pain control, and post-operatively s/p autografting of an extremity. In contrast to the LS, short stretch (SS) bandages have inverse properties. However, they offer similar benefits for edema management and are the standard in lymphedema management. Currently, no literature reports the use of SS in the acute burn setting. This case series aims to describe the use of SS and its observed effects on subjects with acute lower extremity (LE) burns and focuses on its impact on pain control and mobility in the inpatient setting.

**Methods:**

This was a retrospective case series which involved application of SS to the LE to decrease pain and improve mobility by a Certified Lymphedema Therapist (CLT). Data was collected from Jan 2022 to Sept 2024. Data collection included sensation, presence of escharotomy/fasciotomy, total number of SS applications, total duration of SS application, pre/post levels of pain, pre/post levels of mobility (JH-HLM), pre/post ability to ambulate, pre/post mobility assistance levels.

**Results:**

Ten patients were treated with SS, 60% were male with 40% requiring LE escharotomy / fasciotomy. Sensation was intact in 90% and unknown in 1. Dependent pain was reported by 60%. Pre-SS application, 60% were unable to ambulate secondary to pain. Duration of SS treatment averaged 3.9 days (range 1-10), with a mean number of 4.1 (range 2-10) applications. Post-SS, 50 % saw an improvement in the level of assistance required and no patients worsened. Post-SS, 78% saw an improvement in their highest level of mobility. Quantitative data regarding edema was not available in the record, anecdotally no patient’s had worsening of their edema. There were no adverse events related to SS application.

**Conclusions:**

Our findings demonstrate SS application may result in decreased pain and improved mobility with individuals with acute LE burn injuries. No adverse events were reported. Further research is needed to determine the extent of potential benefits of SS application in the acute burn setting.

**Applicability of Research to Practice:**

The ability of SS to provide greater working pressure, in contrast to LS, which have lower working pressure, demonstrates the potential for superior pain and edema control in those with acute burn injuries.

**Funding for the Study:**

N/A

